# Does socioeconomic position affect knowledge of the risk factors and warning signs of stroke in the WHO European region? A systematic literature review

**DOI:** 10.1186/s12889-020-09580-x

**Published:** 2020-09-29

**Authors:** Katie Stack, Wendy Robertson, Clare Blackburn

**Affiliations:** Warwick Medical School, University of Warwick, Coventry, CV4 7HL UK

**Keywords:** Stroke, Socioeconomic position, Knowledge, Awareness, Risk factors, Warning signs, Symptoms, WHO European region, Public health campaigns, Educational interventions

## Abstract

**Background:**

Strokes are one of the leading causes of death worldwide. People with a lower socioeconomic position (SEP) (i.e. with regards to education, income and occupation) are at a higher risk of having a stroke and have worse clinical outcomes compared to the general population. Good knowledge levels about stroke risk factors and warning signs are key to prolonging life and reducing health issues caused by stroke. This systematic review examined differences in knowledge of stroke risk factors and warning signs with regards to SEP in the WHO European region.

**Methods:**

MEDLINE, Embase, Web of Science, PsycINFO and CINAHL were systematically searched using appropriate Medical Subject Headings (MeSH) terms and free text, combining search terms with Boolean operators. Two independent reviewers selected studies in two stages (title and abstract, and full-text), and screened reference lists of included studies. Only studies in English and based in the WHO European region were included.

**Results:**

Screening identified 2118 records. In the final review, 20 articles were included, with 67,309 study participants between them. Out of 17 studies that looked at stroke risk factors, 11 found increasing knowledge to be associated with higher SEP, four found no difference by SEP, one showed a mixed pattern and one outlier study found increasing knowledge of risk factors to be associated with a lower SEP. Out of 19 studies that looked at stroke warning signs or symptoms, 15 found there to be better knowledge of warning signs with a higher SEP, three found there to be no difference, and the same outlier study found increasing knowledge of warning signs with a lower SEP. Studies that seemed to have a higher quality rating found increasing knowledge of stroke with a higher SEP. A meta-analysis was not possible due to heterogeneity of studies.

**Conclusions:**

In the WHO European region, better knowledge of stroke risk factors and warning signs is associated with a higher SEP. Public health campaigns and educational interventions aiming to increase stroke knowledge should be targeted at people with a lower SEP.

## Background

According to The World Health Organisation (WHO), stroke is the second top global cause of death [1]. In Europe, over the last two decades, death rates due to stroke have decreased and currently account for 9% of deaths in men and 13% of deaths in women [2]. The incidence of strokes, however, is expected to increase due to an ageing population [3]. From 2015 to 2035, it has been estimated that there will be a 45% increase in deaths due to stroke and a 34% increase in the total number of stroke events across the European Union [3].

Stroke survivors frequently experience loss of functional, social and financial independence for themselves and their families [4]. Strokes also have a significant economic impact [2]. The total cost of stroke in the European Union in 2015 was estimated as 45 billion euros [3]. Decreasing both the incidence of stroke and associated long-term disability is essential to protect patients and their families but also to reduce the economic burden [3].

Nine out of ten strokes are preventable [5], however, studies suggest that knowledge about risk factors in the population in European countries is sub-optimal [3, 6–8]. Stroke patients who recognise the warning signs of stroke and access treatment early have better outcomes [9, 10]. Evidenced-based public health interventions, to improve knowledge in the European population about stroke risk factors and warning signs, are likely to be key to reducing the burden of strokes.

Inequalities in health knowledge by socioeconomic position (SEP) are important to examine, as systematic reviews looking at sociodemographic factors have found social inequalities in stroke knowledge by gender [11] and by ethnicity [12], with women and white majority populations having better knowledge of stroke risk factors and warning signs compared to men and ethnic minority populations [12]. Although there is extensive literature about the association between SEP and health knowledge, the evidence to date suggests that results vary by health condition [13]. As stroke is a major public health issue, it is important to establish the association between SEP and stroke knowledge. People with a lower SEP have an increased risk of stroke [14] and also have poorer clinical and functional outcomes after having had a stroke [15]. It is important, therefore, to examine differences in stroke knowledge by SEP, as this could increase understanding of why those with a lower SEP are at a higher risk of having poorer outcomes. Scoping of the literature found some studies that showed a positive relationship between higher SEP and better stroke knowledge [16, 17], however other studies showed conflicting results [18, 19]. It is therefore important to do a systematic review of the literature to understand the overall outcome.

The authors are unaware of any systematic reviews analysing stroke knowledge with regards to SEP in the WHO European region. A systematic review was carried out in 2005 looking at the relationship between educational level and stroke knowledge [20]. It has been 15 years, however, since that review was published, and in the interim there has been a significant increase in the literature. There have also been notable public health interventions since 2005, such as the Act FAST campaign [21], which aimed to increase stroke knowledge. To address this important evidence gap, the authors undertook a systematic review, with the aim of providing a synthesis of the available research that addressed knowledge of stroke risk factors and warning signs by SEP in the WHO European region, and assessing the quality of the included studies.

## Methods

The Preferred Reporting Items for Systematic Reviews and Meta-Analyses (PRISMA) checklist [22] was used as a guide whilst carrying out the review.

### Search strategy

A search of the published literature was undertaken on 22/10/2019, using the databases MEDLINE, Embase, Web of Science, PsycINFO and CINAHL (see Additional File 1 for search terms). The databases were systematically searched using appropriate Medical Subject Headings (MeSH) terms and free text, combining search terms with Boolean operators (for example ‘AND’ and ‘OR’). In addition to this, a hand search of the reference lists of included studies, as well as of the similar systematic reviews on gender [11] and ethnic minorities [12] was carried out in order to identify any further studies.

The PEO(S) framework (Population, Exposure of interest, Outcome, Study designs) [23] was used to explore the research question “Does stroke knowledge differ in adults across different SEPs?”. In this paper the term ‘knowledge’ is used. The Oxford Dictionary defines it as “the information, understanding and skills that you gain through education or experience” [24]. Furthermore, the term ‘socioeconomic position’ (SEP) is used as it includes education, income and occupation-based measures [25]. In the database search itself, terms related to SEP were not included. This is because, after doing initial searches, known papers were not always included in the search results if they were not indexed by SEP-related terms. It therefore would have restricted the search results if the authors were to include these terms.

The studies had to fulfil the inclusion criteria listed in Table 1. Although in the medical profession, ‘signs’ and ‘symptoms’ have different meanings [27], the terms were used interchangeably in the search. There was no limit on the publication years for this project, however the search was limited to studies in the English language only.

**Table 1:** Research question and inclusion & exclusion criteria using the PEO(S) framework [23]

PEO(S) components	Inclusion criteria	Exclusion criteria
**P**opulation	• Adult humans (aged 18 years and over) • Countries in the WHO European region [26] (Table 2)	• Children only • Individuals who have had a stroke or transient ischaemic attack (TIA) • Family members / caregivers of stroke patients • Countries outside the WHO European region [26] (Table 2)
**E**xposure of Interest	• Socioeconomic position (education, income and/or occupation-derived measures)	• Studies not analysing at least one aspect of socioeconomic position
**O**utcome	Stroke knowledge: • Risk factors • Warning signs	• Stroke knowledge following an intervention (if no baseline data) • Studies on stroke knowledge but not broken down by warning signs and/or risk factors
**S**tudy Designs	• Cross-sectional studies • Cohort studies • Baseline data of intervention studies (e.g. RCTs, pre-post studies)	• Case studies • Editorials • Qualitative research • Systematic reviews

**Table 2:** Countries in the WHO European region [26]

Albania	Estonia	Lithuania	Serbia
Andorra	Finland	Luxembourg	Slovakia
Armenia	France	Malta	Slovenia
Austria	Georgia	Monaco	Spain
Azerbaijan	Germany	Montenegro	Sweden
Belarus	Greece	Netherlands	Switzerland
Belgium	Hungary	Norway	Tajikistan
Bosnia and Herzegovina	Iceland	Poland	The former Yugoslav Republic of Macedonia
Bulgaria	Ireland	Portugal	Turkey
Croatia	Israel	Republic of Moldova	Turkmenistan
Cyprus	Italy	Romania	Ukraine
Czech Republic	Kyrgyzstan	Russian Federation	United Kingdom
Denmark	Latvia	San Marino	Uzbekistan

### Study selection

The PRISMA flow diagram [22] was used to record the results of the literature search. Two reviewers carried out the study selection independently. Searches of the five electronic databases mentioned above were carried out to identify relevant literature. EndNote, a reference management software package, was used to save the results of the searches and to remove duplicates. The screening stage of the study selection involved reading the title and abstract of each of the articles and excluding those that were not relevant. Rayyan, an online systematic review resource, was used to categorise studies into included and excluded studies. Discrepancies were discussed between the two reviewers. Full text articles of the remaining studies were then obtained and assessed for eligibility, and reasons for article exclusions were justified. The third author was available to discuss any disagreements between the two reviewers. The articles that met the inclusion criteria were included.

### Data extraction

A data extraction tool was devised on an Excel spreadsheet. This included all items required from papers, such as first author, publication year, country, method of cross-sectional study, population, number of respondents, sample size, participant selection, population age range, % women in population, SEP type (e.g. education, income, occupational status), questioning type and results by SEP. This technique facilitated comparisons between studies as well as consistency of data extraction from studies.

### Risk of bias

The critical appraisal tool for cross-sectional studies, AXIS [28], was used to assess the quality of the included studies. This tool includes 20 questions that systematically assess research papers, judge the reliability of studies, and assess the worth and relevance of studies. The questions address the introduction, methods, results, discussion, and other aspects of the studies. This tool received a high level of consensus between medical groups and can be used for systematic reviews [28]. Furthermore, all the studies included in this systematic review were cross-sectional, so it was deemed an appropriate tool to use.

As the measures of SEP, as well as risk factors and warning signs, were too diverse and many studies did not publish data suitable to include in a meta-analysis, a narrative synthesis of the results was carried out, with tables summarising the results of individual studies. The principal summary measures of the results, for the knowledge of risk factors and warning signs of stroke by SEP, included difference in percentage scores, odds ratios with confidence intervals and *p-*values.

## Results

### Study selection

**Fig. 1:**
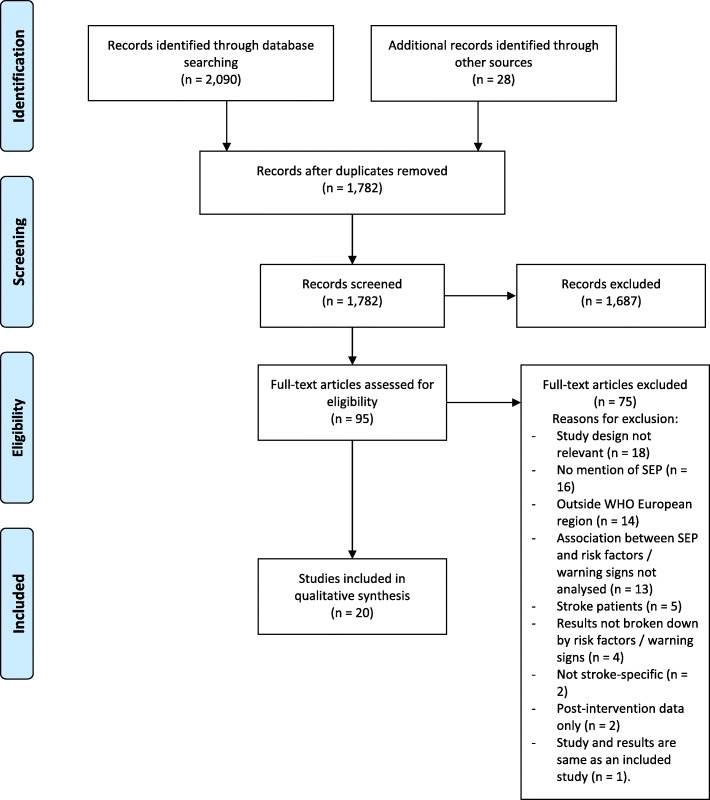
PRISMA Flow Diagram [22] of included studies

The PRISMA flow diagram [22] was used to record the results of the study selection (Fig. 1). The electronic database search identified 2090 records for screening. The reference lists of the final included studies, as well as of the similar systematic reviews on gender [11] and ethnic minorities [12], uncovered a further 28 potentially relevant papers. After duplicates were removed, 1782 studies remained for the title and abstract screen. Of these, 1687 studies were excluded, as they didn’t meet the inclusion criteria, leaving 95 articles for a full text screen to be assessed for eligibility. A further 75 articles were excluded at this stage. There were 20 primary studies that met the inclusion criteria for the review.

### Characteristics of included studies

Characteristics of included studies are displayed in Additional File 2. The 20 included studies [6–8, 16–19, 29–41] were all written between the years 2001 to 2015. Of the 20 studies, four [7, 29–31] were from Spain, two [16, 32] were from Italy and two [33, 34] from Croatia. One study [6] was from both the Republic of Ireland & Northern Ireland. Another study [35] included nine European countries: Austria, France, Germany, Italy, the Netherlands, Poland, Russia, Spain and the UK. The remaining studies included one from each of the following countries: Turkey [36], Israel [37], Portugal [18], Germany [8], France [38], Switzerland [17], Sweden [39], Northern Ireland [40], Denmark [19] and Estonia [41].

All 20 studies [6–8, 16–19, 29–41] were cross-sectional studies. Their methods included telephone interviews [16, 29, 31, 39], questionnaires [7, 8, 18, 19, 32–34, 37, 38, 40, 41] and face-to-face interviews [6, 17, 30, 35, 36]. One article [37] appeared to be a cross-sectional study, however, it seemed to take a longer period to collect data (more than four years).

The overall number of respondents across all 20 studies was 67,309. The number of respondents in the studies ranged from 212 respondents [34] to 28,090 respondents [8]. Participant selection was reported to be random for all studies except one which was snowball (i.e. non-random) [37], one which used consecutive patients [32] and one which was unclear [18]. The sampling strategy for six [7, 17, 33, 34, 38, 41] of the random sampling studies was not clearly reported. The majority of the studies only questioned adults. However, four of them [7, 17, 35, 41] included both adults and children. All studies included both genders; with all except one study [39] having a higher proportion of females.

### Socioeconomic position type and stroke knowledge assessment

Table 3 displays the studies’ characteristics with regards to the aspect of socioeconomic position that results were given by, type of questioning and stroke knowledge. All studies gave results by educational level as a measure of SEP, except one [31] that gave professional status only. Aside from education, two studies also gave results by income [30, 37], one also by both income and professional status [36] and one [40] also by deprivation level.

**Table 3:** Stroke knowledge assessment by aspect of socioeconomic position in WHO European region countries

First author (Date) Country	Socioeconomic position aspect	Open or closed-ended questions (when results were broken down by SEP)?	Knowledge of stroke risk factors?	Knowledge of stroke warning signs?
Baldereschi [16] (2015) Italy	Education	Closed	Y	Y
Dominicis [32] (2006) Italy	Education	Open	Y	Y
Evci [36] (2007) Turkey	Education	Open	Y	Y
	Income			
	Professional status			
Hickey [6] (2009) Republic of Ireland & Northern Ireland	Education	Closed	Y	Y
Lundelin [29] (2012) Spain	Education	Closed	N	Y
Mata [35] (2012) Austria, France, Germany, Italy, the Netherlands, Poland, Russia, Spain and UK	Education	Closed	N	Y
Melnikov [37] (2016) Israel	Education	Open	N	Y
	Income			
Montaner [7] (2001) Spain	Education	Closed	Y	Y
Moreira [18] (2011) Portugal	Education	Closed	Y	Y
Müller-Nordhorn [8] (2006) Germany	Education	Open	Y	N
Neau [38] (2009) France	Education	Open	Y	Y
Nedeltchev [17] (2007) Switzerland	Education	Closed	Y	Y
Nordanstig [39] (2014) Sweden	Education	Open	Y	Y
Parahoo [40] (2003) Northern Ireland	Education	Closed	Y	Y
	Deprivation			
Ramirez-Moreno [30] (2015) Spain	Education	Open	Y	Y
	Income			
Segura [31] (2003) Spain	Professional status	Both	Y	Y
Truelsen [19] (2010) Denmark	Education	Closed	Y	Y
Vibo [41] (2013) Estonia	Education	Closed	Y	Y
Vukovic [33] (2009) Croatia	Education	Closed	Y	Y
Vuletić [34] (2006) Croatia	Education	Closed	Y	Y

Y = yes; N = no

The measures of knowledge of stroke risk factors and warning signs was varied, ranging from open to closed-ended questions. With regards to how studies displayed their type of questioning by SEP, there were 12 studies [6, 7, 16–19, 29, 33–35, 40, 41] that asked closed-ended questions; i.e. the participant was given a list of stroke warning signs and/or risk factors and they were asked to select which ones were correct. This tested their recognition. Seven of the studies [8, 30, 32, 36–39], with regards to SEP, asked open-ended questions, where the participants would be asked to name as many stroke warning signs and/or risk factors as they could. This tested recall. One study [31], with regards to SEP, included a combination of both open and closed-ended questions.

Of the 20 studies, 16 of them [6, 7, 16–19, 30–34, 36, 38–41] included both knowledge of risk factors and warning signs of stroke. One study [8] looked only at risk factors and three [29, 35, 37] looked only at warning signs.

### Risk of bias within studies

Assessment of the quality of the included studies is displayed in Additional File 3, using the AXIS tool for cross-sectional studies [28]. Using this tool, the authors found that the studies were of mixed quality and varied in sampling and response bias.

Only five studies [7, 8, 16, 18, 31] clearly justified their sample size. There were 13 studies [6, 8, 16, 18, 29–33, 35, 36, 39, 40] whose sample frame was taken from an appropriate population base so that it closely represented the target/reference population under investigation. A slightly different set of 13 studies [6, 8, 16, 17, 19, 29–33, 35, 36, 40] used selection processes that were likely to select subjects/participants that were representative of the target/reference population under investigation. Two studies [32, 39] undertook measures to address and categorise non-responders if response rate was low. In all 20 studies [6–8, 16–19, 29–41], the risk factor and outcome variables measured were appropriate to the aims of the study, however only half of the papers [6–8, 17, 18, 30, 35–37, 40] had used measurements/instruments that had been previously trialled, piloted or published. All studies, except three [31, 38, 40], were clear about what was used to determine statistical significance and/or precision estimates. All 20 studies [6–8, 16–19, 29–41] described their methods in sufficient detail to enable them to be repeated.

Basic data were adequately described in the results of all 20 studies [6–8, 16–19, 29–41]. However, in at least nine studies [6, 8, 16, 19, 29–31, 39, 40], the response rate raised concerns about non-response bias. Only one study [38], out of those in which it was relevant, described information about non-response bias. In two studies [32, 36] this was not applicable as the response rate was high. Results were internally consistent for all except three studies [7, 29, 31]. In all 20 studies [6–8, 16–19, 29–41], the results for the analyses described in the methods were presented, and the authors’ discussions and conclusions were justified by the results. Limitations of studies were discussed in all except two studies [36, 40]. No funding sources or conflicts of interest that may have affected the authors’ interpretation of the results were apparent in any of the studies, however in seven studies [7, 31, 33, 34, 38, 40, 41], no information was provided with regards to this. In 11 studies [6, 8, 16, 29, 30, 32, 35–38, 41], ethical approval or consent of participants was attained and in the remaining studies it was unclear.

### Results of individual studies

The most commonly recognised risk factors for stroke across the studies were hypertension, high cholesterol, obesity and smoking (see Additional File 4). Out of eight studies [8, 30–32, 36–39] that asked open questions with regards to risk factors and gave a percentage of the number of people able to give at least one correct risk factor of stroke, the results ranged from 50.8% [32] to 89.5% [38].

The most commonly recognised warning signs of stroke across the studies were weakness of one side of body, speech problems and headache. Out of the eight studies [7, 30–32, 36–39] that asked open questions with regards to stroke warning signs and gave a percentage of the number of people able to give at least one correct warning sign of stroke, the results ranged from 32.6% [31] to 89.1% [37]. Five of these studies [7, 30, 36, 37, 39] gave results over 50%; meaning that over half of participants in these studies were able to correctly identify a warning sign of stroke without being prompted. The other three studies [31, 32, 38] gave results of less than 50%. Out of the five studies [16, 18, 19, 34, 35] that asked closed questions with regards to stroke warning signs and gave a percentage of the number of people able to correctly identify at least one warning sign from a list, the results ranged from 68.7% [16] to 98% [19].

#### Knowledge of stroke risk factors by SEP

Out of the 17 studies [6–8, 16–19, 30–34, 36, 38–41] that assessed knowledge of risk factors for stroke, 11 of them [8, 16, 17, 30–32, 34, 36, 39–41] found there to be better knowledge with a higher SEP (Table 4). With reference to Table 3, all of these studies looked at education except for one article [31], which looked only at professional status, and found that ‘home-based occupations’ (such as housewives, pensioners, unemployed and disabled people) were linked to a lower knowledge of stroke risk factors in comparison with ‘non home-based occupations’ (which included all other occupations). Two of the studies [30, 36], which found this positive association between SEP and risk factor knowledge, also looked at income, and one of these [36] also found this association with professional status.

**Table 4:** Results of individual studies by socioeconomic position

First author (Date) Country	Risk factors			Warning signs		
	No statistically significant differences by SEP?	Statistically better knowledge in higher SEP?	Statistically better knowledge in lower SEP?	No statistically significant differences by SEP?	Statistically better knowledge in higher SEP?	Statistically better knowledge in lower SEP?
Baldereschi [16] (2015) Italy		√			√	
Dominicis [32] (2006) Italy		√^a^			√^a^	
Evci [36] (2007) Turkey		√			√	
Hickey [6] (2009) Republic of Ireland & Northern Ireland	√				√	
Lundelin [29] (2012) Spain	N/A				√	
Mata [35] (2014) Austria, France, Germany, Italy, the Netherlands, Poland, Russia, Spain and UK	N/A				√^a^	
Melnikov [37] (2016) Israel	N/A				√	
Montaner [7] (2001) Spain	√ (for all except arrhythmia)	√ (for arrhythmia)			√	
Moreira [18] (2011) Portugal			√^a^			√^a^
Müller-Nordhorn [8] (2006) Germany		√		N/A		
Neau [38] (2009) France	√			√		
Nedeltchev [17] (2007) Switzerland		√			√^a^	
Nordanstig [39] (2014) Sweden		√			√	
Parahoo [40] (2003) Northern Ireland		√		√		
Ramirez-Moreno [30] (2015) Spain		√			√	
Segura [31] (2003) Spain		√			√	
Truelsen [19] (2010) Denmark	√			√		
Vibo [41] (2013) Estonia		√			√	
Vukovic [33] (2009) Croatia	√				√	
Vuletić [34] (2006) Croatia		√			√	

√ = result of the individual study

^a^= Authors of the individual study reported this result, albeit statistics were not reported. This may be because associations with SEP were not the primary purpose of their study. However, as they reported specific patterns in their results and have reported associations with SEP, the authors of this systematic review have acknowledged these. Please see Additional File 5 for a full quantitative breakdown of results

N/A = not applicable as the study did not look at this aspect. Where cells are empty, this was not a result of the individual study

The authors of one study [7] had disaggregated their results by different risk factors, and found there to be better knowledge of one risk factor (arrhythmia) with higher SEP (*p* < 0.05). However, this same study found no difference with regards to SEP for the other five risk factors (hypertension, diabetes, smoking, alcohol, coronary heart disease (CHD)) [7].

Four studies [6, 19, 33, 38] found there to be no difference in knowledge of stroke risk factors by SEP. For all of these, the measure of SEP was education. Amongst these was one article [33] which found that there was no overall difference, however it found that people with a lower SEP were less likely to name physical inactivity as a risk factor.

Only the authors of one study [18], which had a sample size of 663 respondents, found that there was a higher knowledge of risk factors with a lower SEP; they found that less educated people more frequently recognised stroke risk factors. This was, however, only with regards to vascular risk factors.

#### Knowledge of stroke warning signs by SEP

Out of the 19 studies [6, 7, 16–19, 29–41] that assessed knowledge of stroke warning signs, 15 studies [6, 7, 16, 17, 29–37, 39, 41] found there to be better knowledge of stroke warning signs with a higher SEP (Table 4). With reference to Table 3, all of these articles looked at education, except one [31] which only looked at professional status and found that, similarly as it did for risk factors, ‘home-based occupations’ were associated with a lower knowledge of stroke warning signs. Three of these studies [30, 36, 37] also found this positive association with regards to higher income, and one study [36], found this positive association with regards to professional status. One study [35], which looked at educational level in nine countries, found this positive trend across all countries, however the knowledge level was rather varied between countries. They found that the populations of Austria and Germany were the most knowledgeable, followed by the UK, whilst the populations of Spain and Italy were the least aware of stroke warning signs.

Three studies [19, 38, 40] found there to be no difference in knowledge of warning signs by SEP. One study [18], as it had done for risk factors, found that there was a higher knowledge of warning signs with a lower SEP.

#### Similarities and differences between knowledge of risk factors and warning signs by SEP amongst studies

Out of the 16 studies [6, 7, 16–19, 30–34, 36, 38–41] that included both knowledge of risk factors and warning signs, 12 of them [16–19, 30–32, 34, 36, 38, 39, 41] had similar associations with regards to knowledge of both these factors and SEP. For example, if they found higher SEP to mean better knowledge, they found this for both risk factors and for warning signs. One study [7], as mentioned earlier, found there to be increasing knowledge of warning signs with a higher SEP, but with regards to risk factors there was no difference in knowledge by SEP, except that people of a higher SEP correctly answered arrhythmia as a risk factor.

Two studies [6, 33] found that there was no difference in knowledge of stroke risk factors and level of SEP but that there was a better knowledge of stroke warning signs in higher SEP. One study [40], found the opposite, in that people with a higher SEP had more knowledge of stroke risk factors (*p* < 0.001), but there was no difference in knowledge of warning signs by SEP.

Most of the studies in Spain [29–31, 35] and Italy [16, 32, 35] had similar outcomes in that, where risk factors and warning signs were looked at, increasing knowledge was always positively correlated with a higher SEP. The only exception was a Spanish study which found no difference by SEP with regards to risk factors, except for arrhythmia where it was positively correlated with a higher SEP [7].

The two studies undertaken in Croatia [33, 34] did not have the same correlations with regards to risk factors, but did for warning signs. The studies undertaken in Northern Ireland [6, 40] did not share similar correlations with regards to either risk factors or warning signs, however one of these studies [6] also included the Republic of Ireland and did not distinguish between the two countries with regards to stroke knowledge and its association with SEP. Another study [35] included the UK, amongst other countries, but did not break its results down to show Northern Ireland.

Details of the results of individual studies by SEP are displayed in Additional File 5. Please see this supplementary file for a breakdown of the quantitative results of the individual studies.

### Risk of bias in relation to study results

Although the critical appraisal tool AXIS [28] does not provide a numerical scale for assessing study quality, some studies were found to have answered positively to more questions than others [8, 16, 32, 36] and therefore were of a higher quality. These studies all gave the results of better stroke knowledge with increasing SEP.

Four [31, 34, 37, 41] of the five studies [31, 34, 37, 40, 41], which had the majority of negative or unclear responses using the AXIS tool, and were therefore of a lower quality, found there to be better stroke knowledge with increasing SEP. However, one of these studies [40] found no difference in knowledge of warning signs by SEP.

The other articles that gave results where higher SEP was not necessarily associated with increasing knowledge of stroke [7, 18, 19, 33, 38] had fewer positive responses in the AXIS tool, and therefore were of a lower quality, except for one [6] which had a higher number of positive responses and was therefore of a higher quality.

## Discussion

To the authors’ knowledge this is the first systematic review to report on the association between knowledge of stroke risk factors and warning signs with regards to SEP in the WHO European region. The results indicate that, in general, better stroke knowledge is associated with a higher SEP. Nearly two-thirds of the studies [8, 16, 17, 30–32, 34, 36, 39–41] found that knowledge of stroke risk factors was positively associated with a higher SEP. Only one study [18] found there was a higher knowledge of risk factors with a lower SEP. This study, however, only looked at vascular risk factors. Vascular risk factors are more prevalent amongst people with a lower SEP [42–44], so they may visit their physician more regularly for check-ups. Healthcare professionals are the best source of information on stroke [45] so therefore it’s possible that these patients with a lower SEP could be increasing their knowledge during these visits. Over three quarters of the studies [6, 7, 16, 17, 29–37, 39, 41] found that better knowledge of stroke warning signs was positively associated with a higher SEP. Again, there was only one study that found a higher knowledge with a lower SEP; it was the same one that found higher knowledge of risk factors in lower SEP [18].

The results of this systematic review indicate a social gradient with regards to stroke knowledge. People with a lower SEP had lower levels of both stroke risk factor and warning sign knowledge than those with higher SEPs. Identifying effective interventions to increase stroke knowledge amongst people with lower SEPs is likely to be a key aspect of any strategy to reduce the incidence of stroke in this group and improve stroke outcomes for them. A German study [8] found that sources of information of stroke risk factors varied according to an individual’s socioeconomic profile. It is therefore important to target public health campaigns using platforms and media that people with lower SEPs interact with. Educational interventions have been shown to be effective at increasing stroke warning sign knowledge across all educational groups [46]. More research is needed, however, to identify the extent to which increased knowledge results in positive health behaviour change [3].

Reducing inequities in stroke knowledge amongst people with different SEPs is important for several reasons. Reducing health inequalities in stroke knowledge will make an important contribution to the global aim of health equity [47]. It will also likely contribute to reducing the incidence of stroke and improving outcomes for those with a high risk of stroke. Whilst this review indicates lower stroke knowledge amongst lower SEP groups, it is not clear from the review whether knowledge levels across people with higher SEPs was adequate. The studies in the review all used different definitions of good stroke knowledge and different measures to collect data. In all eight studies [8, 30–32, 36–39] that asked open questions with regards to risk factor knowledge and gave a percentage of the number of people able to give at least one correct risk factor of stroke, over half of the participants in each study could name at least one. When using open questions, risk factor knowledge was better than knowledge of warning signs. However, it could be argued that it is less important to be able to actually recall warning signs; the importance is recognising a stroke when it is occurring [16, 29]. The results of warning sign knowledge were much higher when individuals were given a list to choose from, i.e. when they were asked closed questions rather than open-ended questions. This is more reflective of real life in terms of recognising a stroke. All of the five studies [16, 18, 19, 34, 35], which asked closed questions with regards to stroke warning signs and gave a percentage of the number of people able to correctly identify at least one warning sign from a list, found that over two-thirds of their participants could correctly identify at least one warning sign.

Whilst it is important to increase knowledge levels amongst those with a lower SEP, if knowledge is low overall then it should be increased for all SEP groups. Reviews of various public health domains [48] indicate that mass media campaigns can increase inequality amongst groups by SEP, particularly when directed at the whole population [49]. An example of increasing health inequality through mass media campaigns has been seen amongst ethnic minority groups [50]. The Act FAST public health campaign in the UK [21] aimed to increase knowledge of the signs of stroke amongst the population. However, for BMEs, this campaign had a limited effect [50]. As ethnicity, like SEP, is a social determinant of health outcomes, this example can be used to underline the importance of carefully targeted campaigns. Whilst there are some ethical, economic and practical reasons that can be put forward for targeting people with the lowest SEP [51], the health inequalities literature and evidence suggests that the most effective way to reduce health inequalities is to tackle the social gradient using interventions applied proportionately according to need [52]. As interventions effective in improving outcomes for one socioeconomic group may not be effective for another, tackling the social gradient in stroke knowledge is likely to require interventions carefully targeted to different SEP groups [53].

Carefully targeted social marketing, which uses tailored approaches, may be one effective method to improve knowledge of stroke risk factors and warning signs amongst people with lower SEPs and to narrow the gap between this group and those with higher SEPs, although effects may be small [54]. Educational programmes brought into schools, in order to teach children from a young age, about stroke risk factors and warning signs, may also be of value, particularly in those schools with many children from families with a lower SEP. Events held across Europe for World Stroke Day [55] could be rolled out to areas where there is greater social deprivation and located in areas frequented by people with the poorest knowledge levels.

General Practitioners (GPs) and other primary healthcare professionals have key roles to play in educating patients on stroke awareness and prevention. In OECD countries, people with lower SEPs are less likely to access preventive healthcare from a GP than those with higher SEPs for the same level of need, but once they have seen a GP they have at least as many visits [56]. This suggests that reducing inequities in access to GPs and other primary care professionals for preventative care is important. In addition, using existing primary health care contacts to improve patients’ stroke knowledge and to promote interventions to lower patients’ stroke risk, are likely to be key to reduce the burden of stroke. Risk factor check-ups in workplaces and pharmacies may also be effective [3].

Currently, death rates from stroke vary widely across Europe with poorer stroke outcomes found amongst people in Eastern Europe [3]. In addition to addressing inequalities in stroke risk factor and warning sign knowledge, it is important to address inequalities between European countries, by target efforts to improve stroke knowledge in those countries with the highest death rates and poorest stroke outcome. This is likely to require national and WHO regional strategies across Europe.

This systematic review makes an important contribution to the literature on stroke knowledge. A strength of this review includes the broad search strategy which was used and the comprehensive search of five databases as well as reference list searches of all included articles and of the two similar systematic reviews previously mentioned [11, 12]. The authors used a recognised critical appraisal tool for cross-sectional studies, AXIS [28], to assess the quality of the included studies. A limitation of this tool, however, was that it did not provide a numerical scale for assessing study quality. This meant that there was a degree of subjective assessment required. However, all four [8, 16, 32, 36] of the studies which gave more positive responses to the tools’ questions identified that an increasing knowledge of stroke was associated with increasing SEP. Another limitation was that it was not possible to grade the quality of the evidence and strengths of findings using the AXIS tool, unlike assessment with the GRADE system [57].

The limitations of the study included that a meta-analysis was not possible. This was because the measures of SEP and of knowledge of stroke risk factors and warning signs were too diverse, and many studies did not publish data suitable to include in a meta-analysis. Age ranges of participants were also varied, as well as the use of open-ended or close-ended questions in studies. The implication of different question styles is that different tools will generate different results. Prompting participants with a list of stroke risk factors or warning signs (i.e. closed questions) is different to asking participants to list these items themselves (i.e. open questions). This can affect what ‘good knowledge’ is by what type of questions are asked. Further limitations in this paper include that only studies in the English language were included, and as some studies in other languages may have been relevant, there is potential for publication bias. A further limitation was that grey literature was not included in the search, as the databases used would not have picked this up. However, the authors carried out a hand search of the reference lists of peer-reviewed studies, which would have picked up important grey literature had it been relevant.

Following the study quality appraisal (Additional File 3), it was found that there was a risk of bias amongst some studies, including sampling and selection bias, and concerns about non-response bias. Furthermore, there were several studies which had not had their instruments or measurements for risk factor and outcome variables previously trialled, piloted or published. Those studies, which are at a higher risk of bias, are of a lower quality.

### Implications for future research

As mentioned earlier in the review, there have already been systematic reviews looking at stroke knowledge and its association with gender [11] and ethnic minorities [12]. This review has studied stroke knowledge and its association with socioeconomic position. Another demographic factor which has not been systematically reviewed is age; this could therefore be an important area for future research. Elderly patients are at higher risk of a stroke [58] and may be more likely to witness someone close to them having a stroke, so it is important to understand if they are knowledgeable of the risk factors and warning signs of stroke, and if future interventions also need to be directed towards them. Furthermore, improved education levels amongst young adults may result in greater stroke knowledge amongst this population. However, improving knowledge does not necessarily reduce inequality, as inequality can stay the same albeit with the population as a whole having higher levels of knowledge.

Recognising the warning signs of stroke is important but so is the appropriate response to witnessing them. One study [7] found that extremes of education (i.e. people with no schooling and those with a university degree) would wait longer before acting in the event of a stroke. A review of intent to call an ambulance with regards to SEP would be beneficial. The search also revealed studies that looked at identification of the correct organ affected during a stroke [18, 32, 36]. This could be looked at in another review, as well how people with different SEPs acquire their information. As a similar systematic review found that women had greater stroke knowledge than men [11], it may be important to split interventions by gender and SEP; so that they are targeting specifically men with a lower SEP, in order to reduce inequalities. Systematic reviews to assess effectiveness of public health campaigns across Europe are needed [3], as well as a systematic review of interventions for increasing stroke knowledge for people with lower SEPs, in order to know which interventions are effective.

## Conclusions

The results of this systematic review suggest that individuals living in the WHO European region with a lower socioeconomic position are less knowledgeable of stroke risk factors and less aware of stroke warning signs than those with a higher SEP. As people with a lower SEP are at a higher risk of having a stroke, public health campaigns and educational interventions should be targeted towards them, but in ways that address the social gradient in stroke outcomes and do not further widen inequalities. This would decrease the incidence of stroke and minimise time between stroke onset and treatment, so that loss of life and serious health issues can be minimised, as well as minimising the economic burden caused by stroke.

## Supplementary information

**Additional file 1.** Details of the search strategy. This file provides a detailed description of the search strategy used for finding studies.

**Additional file 2.** Characteristics of included studies. This file provides details of the characteristics of the included studies in a table form, including first author, publication date, country, method of cross-sectional study, population, respondents, participant selection, population age range and % women in the population.

**Additional file 3.** Risk of bias within studies. This file provides details of quality assessment for each of the studies using a critical appraisal tool.

**Additional file 4.** Details of results of individual studies. This file provides details of the breakdown of the results for individual studies.

**Additional file 5.** Details of results of individual studies by socioeconomic position. This file provides details of the breakdown of the results for individual studies by socioeconomic position.

## Data Availability

The datasets supporting the conclusions of this article are included within the article (and its additional files).

## Abbreviations

Approx: Approximately
BME: Black, minority and ethnic
CHD: Coronary heart disease
CI: Confidence intervals
d.f.: Degrees of freedom
DK: Don’t know
F: F-Statistic
FAST: Facial weakness, arm weakness, speech problems, time to call 999
GP: General Practitioner
IFSU: Immigrant from the Former Soviet Union
MeSH: Medical Subject Headings
N: No
N/A: Not applicable
OECD: Organisation for Economic Co-operation and Development
OR: Odds ratio
*P*-value: Probability value
PEO(S): Population, Exposure of interest, Outcome, Study designs
PRISMA: Preferred Reporting Items for Systematic Reviews and Meta-Analyses
RCT: Randomised controlled trial
Ref: Reference category
RF: Risk factor
SD: Standard deviation
SEP: Socioeconomic position
TIA: Transient ischaemic attack
VR: Veteran Resident
WHO: World Health Organisation
WS: Warning sign
Y: Yes
Yrs: years
