# Supplementary material for: Does socioeconomic position affect knowledge of the risk factors and warning signs of stroke in the WHO European region? A systematic literature review
Source: BMC Public Health. 2020 Sep 29;20:1473. doi: 10.1186/s12889-020-09580-x (PMC7526368; doi:10.1186/s12889-020-09580-x)
Supplement: Supplementary file 1 — Additional file 1. Details of the search strategy. This file provides a detailed description of the search strategy used for finding studies. [file 12889_2020_9580_MOESM1_ESM.docx]

**Additional File 1: Details of the search strategy**

The following five electronic databases were searched on 22/10/2019:

1. MEDLINE
2. Embasne
3. Web of Science
4. PsycINFO
5. CINAHL

The search strategies for each are presented below, in the same format as how they were displayed on each database.

1. MEDLINE (414 results)

| **#** | **Searches** | **Results** |
| --- | --- | --- |
| 1 | exp Health Knowledge, Attitudes, Practice/ | 105,723 |
| 2 | exp Awareness/ | 19,380 |
| 3 | 1 or 2 | 122,858 |
| 4 | exp “Signs and Symptoms”/ | 2,008,656 |
| 5 | exp Risk Factors/ | 787,428 |
| 6 | 4 or 5 | 2,690,961 |
| 7 | exp Stroke/ | 126,224 |
| 8 | cerebrovascular accident.mp. | 3,791 |
| 9 | 7 or 8 | 128,929 |
| 10 | 3 and 6 and 9 | 446 |
| 11 | limit 10 to english language | 414 |

1. Embase (876 results)

| **#** | **Searches** | **Results** |
| --- | --- | --- |
| 1 | exp knowledge/ | 162,229 |
| 2 | exp attitude to health/ | 110,319 |
| 3 | exp awareness/ | 77,846 |
| 4 | 1 or 2 or 3 | 233,813 |
| 5 | sign.mp. | 154,848 |
| 6 | exp symptom/ | 147,334 |
| 7 | exp risk factor/ | 986,323 |
| 8 | 5 or 6 or 7 | 1,269,368 |
| 9 | exp cerebrovascular accident/ | 202,550 |
| 10 | stroke.mp. | 431,698 |
| 11 | 9 or 10 | 488,458 |
| 12 | 4 and 8 and 11 | 922 |
| 13 | limit 12 to english language | 876 |

1. Web of Science (156 results)

| **Set** | **Results** |  |
| --- | --- | --- |
| #4 | 156 | #3 AND #2 AND #1  *Indexes=SCI-EXPANDED, SSCI Timespan=All years* |
| #3 | 203,027 | (TS=(“cerebrovascular accident” or stroke*)) *AND* LANGUAGE: (English) *AND* DOCUMENT TYPES: (Article)  *Indexes=SCI-EXPANDED, SSCI Timespan=All years* |
| #2 | 1,480,317 | (TS=(signs or sign or symptom* or “risk factor*”)) *AND* LANGUAGE: (English) *AND* DOCUMENT TYPES: (Article)  *Indexes=SCI-EXPANDED, SSCI Timespan=All years* |
| #1 | 23,267 | (TS=((knowledge or awareness) NEAR/6 health)) *AND* LANGUAGE: (English) *AND* DOCUMENT TYPES: (Article)  *Indexes=SCI-EXPANDED, SSCI Timespan=All years* |

1. PsycINFO (354 results)

| **#** | **Searches** | **Results** |
| --- | --- | --- |
| 1 | exp Health Knowledge/ | 7,465 |
| 2 | exp Awareness/ | 87,752 |
| 3 | 1 or 2 | 94,974 |
| 4 | sign*.mp. | 1,139,766 |
| 5 | exp Symptoms/ | 230,523 |
| 6 | exp Risk Factors/ | 76,277 |
| 7 | 4 or 5 or 6 | 1,328,207 |
| 8 | exp Cerebrovascular Accidents/ | 20,174 |
| 9 | stroke.mp. | 33,709 |
| 10 | 8 or 9 | 35,136 |
| 11 | 3 and 7 and 10 | 363 |
| 12 | limit 11 to english language | 354 |

1. CINAHL (290 results)

| **Search ID#** | **Search Terms** | **Results** |
| --- | --- | --- |
| S6 | S3 AND S4 AND S5 | 290 |
| S5 | (MH “Stroke+”) | 61,636 |
| S4 | (MH “Risk Factors+”) OR (MH “Signs and Symptoms+”) | 738,633 |
| S3 | S1 OR S2 | 113,701 |
| S2 | “awareness” | 60,665 |
| S1 | (MH “Health Knowledge”) OR (MH “Knowledge+”) | 58,070 |

In addition to the electronic database search, a hand search of the reference lists of the final included studies, as well as of the two similar systematic reviews previously mentioned, based on gender (11) and ethnic minorities (12), was carried out.
