# Supplementary material for: Does socioeconomic position affect knowledge of the risk factors and warning signs of stroke in the WHO European region? A systematic literature review
Source: BMC Public Health. 2020 Sep 29;20:1473. doi: 10.1186/s12889-020-09580-x (PMC7526368; doi:10.1186/s12889-020-09580-x)
Supplement: Supplementary file 2 — Additional file 2. Characteristics of included studies. This file provides details of the characteristics of the included studies in a table form, including first author, publication date, country, method of cross-sectional study, population, respondents, participant selection, population age range and % women in the population. [file 12889_2020_9580_MOESM2_ESM.docx]

**Additional file 2: Characteristics of included studies**

| **First author (Date) Country** | **Method of cross-sectional study** | **Population** | **Number of respondents / Sample size (Response rate %)** | **Participant selection** | **Population age range (years)** | **Women in population (%)** |
| --- | --- | --- | --- | --- | --- | --- |
| **Baldereschi^(16)^ (2015) Italy** | Telephone survey | Residents aged ≥18 | 1,000 / 9,863 (10%) | Random stratified sampling | 18-80 | 51.9 |
| **Dominicis^(32)^ (2006) Italy** | Semi-structured questionnaire | Hospital ultrasound department outpatients | 352 / 357 (99%) | Consecutive outpatients referred to hospital | No range given. Median age of 67 yrs. | 52.3 |
| **Evci^(36)^ (2007) Turkey** | Face-to-face interview | People aged >40 years registered at urban health centres | 920 / 920 (100%) | Random cluster sampling | >40.  Mean age 53.5 yrs (±10.46). | 59.9 |
| **Hickey^(6)^ (2009) Republic of Ireland & Northern Ireland** | Home interview | Community-dwelling 65+ year olds | 2,033 respondents. 68% response rate. | Random selection using most complete population listings | 65-102 | 57.0 |
| **Lundelin^(29)^ (2012) Spain** | Telephone interview | Spanish non-instutionalised adults | 11,827 respondents. 55% response rate. | Random using multistage cluster sampling | 18-65+ | No data given. |
| **Mata^(35)^ (2014) Austria, France, Germany, Italy, the Netherlands, Poland, Russia, Spain and UK** | Face-to-face interview | 14-98 year olds in nine European countries | 10,228 respondents | Random systematic and quota sampling | 14–98 | 52.2 |
| **Melnikov^(37)^ (2016) Israel** | Questionnaire | Interviewers’ friends & family members, aged ≥40 years with no history of stroke | 643 respondents | Non-random (snowball) | ≥40 | 59.3 |
| **Montaner^(7)^ (2001) Spain** | Questionnaire | Visitors from primary health centres | 935 / 1,050 (89%) | Random (sampling method not clear) | Unclear but includes <25yrs to >65yrs | 62.1 |
| **Moreira^(18)^ (2011) Portugal** | Questionnaire | 1% sample of adult health centre users registered at three community health centres | 663 respondents | Unclear | ≥18 | 70.3 |
| **Müller‑Nordhorn^(8)^ (2006) Germany** | Questionnaire | Residents of Berlin aged ≥50 years | 28,090 / 75,720 (37%) | Random selection from whole population | ≥50 | 56.5 (approx. – some data missing) |
| **Neau^(38)^ (2009) France** | Questionnaire | Adults living in Poitou-Charentes | 411 / 437 (94%) | Random (sampling method not clear) | 18-70+ | 54.3 |
| **Nedeltchev^(17)^ (2007) Switzerland** | Face-to-face interview | People on the streets of Bern | 422 respondents | Random (sampling method not clear) | All ages | 58.0 |
| **Nordanstig^(39)^ (2014) Sweden** | Telephone interview | Residents of Sweden aged 18–79 years | 1,500 / 2,423 (62%) | Random stratified sampling | 18-79 | 47.0 |
| **Parahoo^(40)^ (2003) Northern Ireland** | Self-completed questionnaire | Northern Irish population | 892 / 2,000 (45%) | Multi-stage systematic random sampling | 18-65+ | 58.6 |
| **Ramirez-Moreno^(30)^ (2015) Spain** | Face-to-face interview | Adults residing in Extremadura who had no cognitive impairment | 2,411 / 3,342 (72%) | Multi-stage random sampling | ≥18 | 59.8 |
| **Segura^(31)^ (2003) Spain** | Telephone interview | Adults in Spain | 2,884 / 3,000 (96%) | Multi-stage random sampling using quotas | ≥18 | 51.6 |
| **Truelsen^(19)^ (2010) Denmark** | Web-based questionnaire | Danish population aged 40+ years | 811 / 3,520 (23%) | Random stratified sample from a study panel | 40-70+ | 50.0 |
| **Vibo^(41)^ (2013) Estonia** | Questionnaire | Estonian-speaking subjects selected in public places of two big cities (Tallinn and Tartu) | 355 respondents | Random (sampling method not clear) | 10-60+ | 69.0 |
| **Vukovic^(33)^ (2009) Croatia** | Questionnaire | Adults attending outpatient services at Department of Neurology | 720 / 1,000 (72%) | Random (sampling method not clear) | 18-60+ | 54.9 |
| **Vuletić^(34)^ (2006) Croatia** | Questionnaire | Symptom-free adults | 212 respondents | Random (sampling method not clear) | 18-60+ | 55.0 |
