# Supplementary material for: Does socioeconomic position affect knowledge of the risk factors and warning signs of stroke in the WHO European region? A systematic literature review
Source: BMC Public Health. 2020 Sep 29;20:1473. doi: 10.1186/s12889-020-09580-x (PMC7526368; doi:10.1186/s12889-020-09580-x)
Supplement: Supplementary file 3 — Additional file 3. Risk of bias within studies. This file provides details of quality assessment for each of the studies using a critical appraisal tool. [file 12889_2020_9580_MOESM3_ESM.docx]

**Additional File 3: Risk of bias within studies using critical appraisal AXIS tool for cross-sectional studies (28)**

| **QUESTION** | **INTRODUCTION: 1) Were the aims/objectives of the study clear?** | **METHODS: 2) Was the study design appropriate for the stated aim(s)?** | 1. **Was the sample size justified?** | 1. **Was the target/reference population clearly defined? (Is it clear who the research was about?)** | 1. **Was the sample frame taken from an appropriate population base so that it closely represented the target/reference population under investigation?** | 1. **Was the selection process likely to select subjects/participants that were representative of the target/reference population under investigation?** | 1. **Were measures undertaken to address and categorise non-responders?** | 1. **Were the risk factor and outcome variables measured appropriate to the aims of the study?** | 1. **Were the risk factor and outcome variables measured correctly using instruments/measurements that had been trialled, piloted or published previously?** | 1. **Is it clear what was used to determine statistical significance and/or precision estimates? (e.g. p values, CIs)** | 1. **Were the methods (including statistical methods) sufficiently described to enable them to be repeated?** | **RESULTS: 12) Were the basic data adequately described?** | 1. **Does the response rate raise concerns about non-response bias?** | 1. **If appropriate, was information about non-responders described?** | 1. **Were the results internally consistent?** | 1. **Were the results for the analyses described in the methods, presented?** | **DISCUSSION: 17) Were the authors' discussions and conclusions justified by the results?** | 1. **Were the limitations of the study discussed?** | **OTHER: 19) Were there any funding sources or conflicts of interest that may affect the authors' interpretation of the results?** | 1. **Was ethical approval or consent of participants attained?** |
| --- | --- | --- | --- | --- | --- | --- | --- | --- | --- | --- | --- | --- | --- | --- | --- | --- | --- | --- | --- | --- |
| **FIRST AUTHOR** |  |  |  |  |  |  |  |  |  |  |  |  |  |  |  |  |  |  |  |  |
| **Baldereschi^(16)^** | Y | Y | Y | Y | Y | Y | N | Y | N | Y | Y | Y | Y | N | Y | Y | Y | Y | N | Y |
| **Dominicis^(32)^** | Y | Y | N | Y | Y | Y | Y | Y | N | Y | Y | Y | N | N/A | Y | Y | Y | Y | N | Y |
| **Evci^(36)^** | Y | Y | N | Y | Y | Y | N/A | Y | Y | Y | Y | Y | N | N/A | Y | Y | Y | N | N | Y |
| **Hickey^(6)^** | Y | Y | N | Y | Y | Y | N | Y | Y | Y | Y | Y | Y | N | Y | Y | Y | Y | N | Y |
| **Lundelin^(29)^** | Y | Y | N | Y | Y | Y | N | Y | N | Y | Y | Y | Y | N | N | Y | Y | Y | N | Y |
| **Mata^(35)^** | Y | Y | N | Y | Y | Y | N | Y | Y | Y | Y | Y | DK | N | Y | Y | Y | Y | N | Y |
| **Melnikov^(37)^** | Y | Y | N | Y | N | N | N | Y | Y | Y | Y | Y | DK | N | Y | Y | Y | Y | N | Y |
| **Montaner^(7)^** | Y | Y | Y | Y | N | N | N | Y | Y | Y | Y | Y | N | N | N | Y | Y | Y | DK | DK |
| **Moreira^(18)^** | Y | Y | Y | Y | Y | N | N | Y | Y | Y | Y | Y | DK | N | Y | Y | Y | Y | N | DK |
| **Müller-**  **Nordhorn^(8)^** | Y | Y | Y | Y | Y | Y | N | Y | Y | Y | Y | Y | Y | N | Y | Y | Y | Y | N | Y |
| **Neau^(38)^** | Y | Y | N | Y | N | N | N | Y | N | N | Y | Y | N | Y | Y | Y | Y | Y | DK | Y |
| **Nedeltchev^(17)^** | Y | Y | N | Y | N | Y | N | Y | Y | Y | Y | Y | DK | N | Y | Y | Y | Y | N | DK |
| **Nordanstig^(39)^** | Y | Y | N | Y | Y | N | Y | Y | N | Y | Y | Y | Y | N | Y | Y | Y | Y | N | DK |
| **Parahoo^(40)^** | Y | Y | N | Y | Y | Y | N | Y | Y | N | Y | Y | Y | N | Y | Y | Y | N | DK | DK |
| **Ramirez-Moreno^(30)^** | Y | Y | N | Y | Y | Y | N | Y | Y | Y | Y | Y | Y | N | Y | Y | Y | Y | N | Y |
| **Segura^(31)^** | Y | Y | Y | Y | Y | Y | N | Y | N | N | Y | Y | Y | N | N | Y | Y | Y | DK | DK |
| **Truelsen^(19)^** | Y | Y | N | Y | N | Y | N | Y | N | Y | Y | Y | Y | N | Y | Y | Y | Y | N | DK |
| **Vibo^(41)^** | Y | Y | N | Y | N | N | N | Y | N | Y | Y | Y | DK | N | Y | Y | Y | Y | DK | Y |
| **Vukovic^(33)^** | Y | Y | N | Y | Y | Y | N | Y | N | Y | Y | Y | N | N | Y | Y | Y | Y | DK | DK |
| **Vuletić^(34)^** | Y | Y | N | Y | N | N | N | Y | N | Y | Y | Y | DK | N | Y | Y | Y | Y | DK | DK |

Y = yes; N = no; DK = don’t know; N/A = not applicable.
