# Supplementary material for: Does socioeconomic position affect knowledge of the risk factors and warning signs of stroke in the WHO European region? A systematic literature review
Source: BMC Public Health. 2020 Sep 29;20:1473. doi: 10.1186/s12889-020-09580-x (PMC7526368; doi:10.1186/s12889-020-09580-x)
Supplement: Supplementary file 4 — Additional file 4. Details of results of individual studies. This file provides details of the breakdown of the results for individual studies. [file 12889_2020_9580_MOESM4_ESM.docx]

**Additional File 4: Details of results of individual studies**

| **First author (Date) Country** | **Open or closed-ended questions?** | **Knowledge of stroke risk factors** | **Knowledge of stroke warning signs** |
| --- | --- | --- | --- |
| **Baldereschi^(16)^ (2015) Italy** | Closed | 51.3% of respondents recognised more than one risk factor.  14.6% of respondents recognised more than two risk factors.  Risk factors were recognised in the following percentages:  Hypertension = 67.6%  High cholesterol = 44.0%  Family history of stroke = 16.0%  Obesity = 15.1%  Diabetes = 13.3%  Heart diseases = 8.0%  Age = 5.4% | 31.3% could not identify any stroke warning sign, meaning 68.7% could identify at least one sign.  Stroke symptoms were recognised in the following percentages:  Sudden weakness of arm and/or leg on one side of the body = 68.7%  Sudden confusion or trouble speaking = 58.8%  Sudden trouble seeing or blindness = 11.0%  Incorrect options = 13.9% |
| **Dominicis^(32)^ (2006) Italy** | Open | 50.8% of subjects had knowledge of at least one correct risk factor for stroke.  Risk factors responses were given in the following percentages when asked open question:  “I don’t know” = 40.1%  Hypertension = 25.6%  High cholesterol = 17.3%  Smoking = 14.2%  Stress, emotional factors = 11.7%  Alcohol abuse = 9.7%  Bad diet = 8.2%  Other responses = 11.1% | 47.7% of subjects had knowledge of at least one correct symptom of stroke.  Stroke onset symptom answers were given in the following percentages when asked open question:  “I don’t know” = 48.6%  Headache = 12.2%  Unilateral weakness (any) = 11.4%  Vertigo, dizziness, poor balance = 10.5%  Syncope, loss of consciousness = 8.2%  Sensory symptoms = 5.1%  Speech disorder = 4.5%  Chest pain = 2.0%  Other responses = 7.7% |
| **Evci^(36)^ (2007) Turkey** | Open | 63.6% of participants knew at least one risk factor of stroke.  Risk factors responses were given in the following percentages when asked open question:  Stress = 34.9%  Hypertension = 34.1%  Diabetes mellitus = 9.8%  High cholesterol = 6.5%  Smoking = 5.0%  Obesity = 3.7%  Older age = 1.1%  Heart disease = 3.0%  Alcohol use = 0.9%  Genetic tendency = 0.6%  Physical inactivity = 0.4% | 79.9% of participants knew at least one warning sign for stroke.  Warning signs were given in the following percentages when asked open question:  Paralysis/weakness at one side of face and body = 37.1%  Trouble in speaking or understanding = 26.8%  Numbness at one side of face and body = 14.2%  Unconsciousness = 10.4%  Visual problems = 6.8%  Vertigo = 3.5%  Severe headache = 0.6%  Difficulty in understanding = 0.5%  Difficulty in breathing = 0.1% |
| **Hickey^(6)^ (2009) Republic of Ireland & Northern Ireland** | Closed | 6% of participants were unable to identify any stroke risk factors (it is not clear if all of the remaining 94% correctly identified at least one risk factor).  Risk factor responses:  Hypertension* = 75%  Stress = 43%  Cholesterol* = 40%  Smoking* = 30%  Obesity = 30%  Lack of exercise = 18%  Family history of stroke = 16%  Diabetes* = 11%  Alcohol use* = 10%  No response/don't know = 6%  *Established risk factor | 13% of participants identified no warning signs of stroke (it is not clear if the remaining 87% knew at least one correct warning sign).  Warning sign responses:  Slurred speech* = 54%  Dizziness* = 44%  Numbness (any)* = 41%  Weakness (any)* = 38%  Headache* = 29%  Vision problems* = 20%  Difficulty understanding = 18%  No response/don't know = 13%  *Established warning sign |
| **Lundelin^(29)^ (2012) Spain** | Closed | N/A | Each of the five main stroke symptoms was identified by at least three quarters of the respondents.  Exact percentages of responses for each symptom are difficult to interpret from the results, however the following options were given for the respondents to choose from:  Sudden confusion or trouble speaking  Numbness or weakness of face, arm or leg  Sudden chest pain (not a stroke symptom)  Severe headache with no known cause  Sudden trouble seeing in one or both eyes  Sudden trouble walking, dizziness, or loss of balance.  No data given with regards to % of participants able to recognise at least one warning sign. |
| **Mata^(35)^ (2014) Austria, France, Germany, Italy, the Netherlands, Poland, Russia, Spain and UK** | Closed | N/A | Of 14 stroke symptoms, none of them were recognised by more than 50% of participants.  19% of participants could not identify any symptom, meaning 81% could recognise at least one symptom.  Percentage of participants who recognised a condition as a symptom for a stroke:  Slurred speech = 44.0%  Paralysis = 43.0%  Lopsided face = 38.3%  Dizziness = 32.5%  Inclination to fall to one side = 29.8%  Sudden confusion = 26.2%  Prickly feeling = 24.7%  Numbness = 23.2%  Debility = 18.9%  Sudden one-sided blindness = 17.9%  Spit running out of mouth = 15.4%  Problems eating = 9.5%  Earache = 5.9% (not a typical stroke symptom)  Frequent difficulties swallowing = 5.6%  Runny eyes = 4.0%  I don’t know = 19.1% |
| **Melnikov^(37)^ (2016) Israel** | Open | 11.2% of respondents did not know any stroke risk factors, meaning 88.8% could name at least one risk factor.  Risk factor answers given by respondents on open questioning:  Don’t know any risk factor = 12.6% of Veteran Residents (VRs); 9.5% of Immigrants from the Former Soviet Union (IFSUs) (overall average = 11.2%)  Hypertension = 50.4% VRs; 62.8% IFSUs  High cholesterol = 34.3% VRs; 33.2% IFSUs  Smoking = 26.9% VRs; 27.4% IFSUs  Obesity = 22.1% VRs; 20.2% IFSUs  Diabetes = 17.6% VRs; 19.7% IFSUs  Stress = 14.8% VRs; 15.2% IFSUs  Heart disease = 10.2% VRs; 12.1% IFSUs  Sedentary lifestyle = 9.0% VRs; 6.7% IFSUs  Family history = 10.0% VRs: 4.9% IFSUs  Poor diet = 7.6% VRs; 6.3% IFSUs  Old age = 5.5% VRs; 8.1% IFSUs  Previous stroke = 0.7% VRs; 0.4% IFSUs  N.B. Risk factor results were given but were not broken down by SEP, so are not displayed in Additional file 5. | 10.9% of respondents did not know any stroke warning signs, meaning 89.1% could name at least one warning sign.  The respondents were requested to provide three signs or symptoms of stroke with which they were familiar. The mean number of stroke warning signs reported was 2.1 for Immigrants from the Former Soviet Union (IFSUs) and 2.0 for Veteran Residents (VRs).  Warning sign answers given by respondents on open questioning:  Don’t know any warning sign = 11.4% VRs; 9.9% IFSUs (overall average = 10.9%)  Sudden weakness or paralysis on one side of the body = 49.8% VRs; 53.4% IFSUs  Trouble speaking = 32.9% VRs; 39.5% IFSUs  Sudden confusion = 21.7% VRs; 17.5% IFSUs  Sudden severe headache = 18.8% VRs; 23.7% IFSUs  Sudden numbness on one side of the body = 10.7% VRs; 9.0% IFSUs  Loss of balance or coordination or sudden trouble walking = 10.7% VRs; 13.0% IFSUs  Sudden trouble seeing = 10.6% VRs; 8.7% IFSUs  Dizziness = 8.4% VRs; 13.7% IFSUs  Sudden chest or arm pain = 5.5% VRs; 1.8% IFSUs (not a stroke warning sign)  Epilepsy or spasm = 3.8% VRs; 3.1% IFSUs  Sudden breathing problems = 1.0% VRs; 1.4% IFSUs  Other = 34.0% VRs; 35.9% IFSUs. |
| **Montaner^(7)^ (2001) Spain** | Closed for risk factors; Both for warning signs | 5.5% of people polled identified all six of the risk factors.  37% of the population polled had a good knowledge of risk factors (defined as knowing five or more and make one error or less).  Risk factors were recognised in the following percentages:  High blood pressure = 95.4%  Alcoholism = 83.1%  Tobacco = 80.3%  Previous cardiac infarction = 68.7%  Arrhythmia = 55.1%  Diabetes = 55.1%  No data given with regards to % of participants able to recognise at least one risk factor. | On being asked openly if they knew any of the stroke warning signs, those polled spontaneously mentioned paresthesias and hemiparesis mostly (55.4%) and 27.2% of the population was not able to mention any of them. This means that 72.8% were able to name at least one warning sign.  When the signs were listed, 12.9% of people polled identified all five of the correct signs and none of the incorrect ones. 50% of the population polled had a good knowledge of signs and symptoms of stroke (defined as those who knew three or more warning signs of the list and made one error or less).  The signs and symptoms when listed, were recognised in the following percentages:  Paresthesias and hemiparesis = 94.9%  Aphasia-dysarthria = 93.2%  Loss of vision = 78.9%  Headache = 78.6%  Vertigo = 68.9% |
| **Moreira^(18)^ (2011) Portugal** | Closed | Risk factors identified:  High blood pressure = 86.0%  High cholesterol = 78.4%  Overweight = 66.5%  Alcohol abuse = 64.9%  Smoking = 61.7%  No physical exercise = 59.1%  Heart condition = 56.7%  Stress = 56.6%  Diabetes = 55.8%  Unhealthy diet = 45.2%  Family history of stroke = 40.7%  Sedentary lifestyle = 36.8%  Arrhythmia = 33.5%  No data given with regards to number of stroke risk factors recognised individually. | Half of the participants identified at least seven out of the 19 symptoms listed. Overall, 39.7% of participants recognised simultaneously the three key warning signs – hemibody paralysis, sudden difficulty in speaking/understanding/writing and weakness/difficulty in movements of arm/leg/face; 17.6% did not recognise any of them. This means that 82.4% of participants were able to recognise at least one warning sign.  Warning signs identified:  Paralysis of one side of the body = 64.1%  Speech/language disturbances = 60.6%  Weakness/difficulty in moving arm/leg or face = 60.5%  Imbalanced gait = 50.7%  Paralysis of any part of the body = 50.1%  Vestibular symptoms (dizziness/vertigo) = 47.7%  Sensory symptoms (numbness in part of the body) = 47.4%  Faintness = 45.4%  Chest pain = 39.4% (not stroke warning sign)  Confusion (disorientation) = 38.3%  Blurred vision = 36.2%  Loss of vision in one eye (whole or part) = 33.8%  Generalised weakness (unexplained falls) = 30.0%  Shortness of breath = 27.0% (not stroke warning sign)  Difficulty in swallowing = 20.1%  Double vision = 18.6%  Incontinence of urine or faeces = 16.6%  Pain (no specific location) = 16.0% (not stroke warning sign)  Tinnitus = 14.0%. |
| **Müller‑Nordhorn ^(8)^ (2006) Germany** | Open | 68% of respondents were able to correctly name at least one stroke risk factor (with 10% naming one, 20% naming two, 25% naming three, and 13% the maximum of four correct risk factors).  Stroke risk factors named in open-ended questions by survey respondents:  Hypertension = 43%  Smoking = 39%  Obesity = 34%  Physical inactivity = 20%  Stress = 18% (not an established risk factor)  Alcohol consumption = 16%  Poor diet/nutrition = 14%  Hypercholesterolaemia = 13%  Diabetes = 8%  Family history of stroke = 3%  Heart disease = 2%  Age = 1%  Male sex = 0% | N/A |
| **Neau^(38)^ (2009) France** | Open | 62.3% of respondents were able to name three risk factors, 18.2% quoted two risk factors, 8.5% one, and 10.5% were unable to correctly recognise one risk factor or said that they did not know even a single stroke risk factor. This meant that 89.5% of respondents were able to correctly name a risk factor.  Respondent’s knowledge of stroke risk factors on open questioning:  Smoking = 48.4%  Alcohol = 37.7%  High cholesterol = 29.7%  Bad diet = 25.8%  High blood pressure = 20.9%  Stress = 17.5%  Obesity = 13.1%  Lack of exercise = 12.4%  Cardiovascular diseases = 8.8%  Diabetes = 8.5%  Drug abuse = 3.2%  Heredity = 2.9%  Age = 2.2%  Oral contraceptives = 1.5%  Others = 15.1% | 49.9% of respondents were able to recognise one or more stroke warning sign.  50.1% responded that they did not know anything or were unable to give pertinent information.  Respondents’ knowledge of stroke warning signs on open questioning:  Loss of consciousness = 41.6%  Weakness, paralysis = 29.9%  Difficulty in speaking reading or comprehension = 18.5%  Severe headache = 15.6%  Weakness, paralysis on one side of the body = 14.4%  Blurred, double or loss of vision = 11.4%  Dizziness (vertigo), loss of balance = 10.7%  Respiratory disturbances = 7.5%  Pain = 5.6%  Sensory deficit = 2.9%  Other = 34.5% |
| **Nedeltchev^(17)^ (2007) Switzerland** | Closed | Good knowledge of stroke risk factors (being able to name five or more and make one error or less) was noted in 6.4% of surveyed persons.  No data given with regards to % of surveyed persons able to correctly recognise at least one risk factor and no results breakdown of risk factors given. | Good knowledge of stroke warning signs (correctly recognising three or more symptoms of the list and making one error or less) was noted in 64% of surveyed persons.  No data given with regards to % of surveyed persons able to correctly recognise at least one warning sign and no results breakdown of warning signs given. |
| **Nordanstig^(39)^ (2014) Sweden** | Open | 86% named at least one correct risk factor, 69% at least two, and 46% correctly listed three or more.  Knowledge of stroke risk factors on open questioning:  Smoking = 40%  Stress = 30%  Hypertension = 28%  Obesity = 23%  Unhealthy diet = 23%  Lack of exercise = 19%  Alcohol = 11%  High cholesterol = 9%  Family history of stroke = 8%  Cardiovascular disease = 5%  Diabetes = 3%  Old age = 3%  Depression = 0.5%  Previous stroke = 0.2%  Salt intake = 0.1% | 72% could correctly report at least one stroke symptom, 43% at least two, and 13% three or more.  Knowledge of stroke symptoms on open questioning:  Difficulty understanding or slurred speech = 39%  Trouble walking/dizziness/loss of balance = 24%  Numbness = 22%  Weakness = 22%  Headache = 14%  Vision problem = 7% |
| **Parahoo^(40)^ (2003) Northern Ireland** | Both for risk factors; Closed for warning signs | Respondents’ recognition of risk factors:  High blood pressure = 94.4%  Previous stroke = 94.3%  Family history = 83.5%  Cigarette smoking = 83.9%  High cholesterol level = 76.3%  Disease of the arteries = 66.7%  Stress = 66.6%  Obesity = 66.2%  Advancing age = 62.5%  Excessive alcohol intake = 60.7%  Lack of exercise = 60.4%  Heart disease = 51.4%  Diabetes = 30.5%  Men rather than women = 19.3%  No data on number of correctly recognised risk factors.  When asked in an open-ended question to identify the main risk factor, 36.1% correctly identified hypertension, with a further 23.9% considering smoking to be the main risk factor. Almost all of the remaining respondents identified an ‘unhealthy lifestyle’ such as poor diet and lack of exercise as the main risk factor of stroke. | Respondents’ recognition of warning symptoms:  Sudden weakness of arm and leg on one side of the body = 92.2%  Sudden loss of speech or inability to express oneself in words = 89.9%  Sudden onset of vertigo, imbalance and clumsiness of limbs = 47.6%  Sudden loss of vision in one eye = 41.6%  Sudden onset of unconsciousness with generalised convulsion and incontinence = 30.5%  Frequent headache with flashing lights and nausea = 25.0%  Sudden onset of chest pain with breathlessness and sweating = 23.7%  Sudden severe headache with vomiting and neck stiffness = 15.8%  Inability to walk due to sudden onset of backache = 6.3%  Sudden loss of appetite with abdominal pain, nausea and diarrhoea = 2.6%  No data on number of correctly recognised warning signs. |
| **Ramirez-Moreno^(30)^ (2015) Spain** | Open | 40.8% of respondents were unable to list any vascular risk factors related to stroke and the remaining 59.2% were able to list at least one risk factor.  Perception of stroke risk factors among survey respondents (open-ended questions):  Smoking = 50.5%  High blood pressure = 49.9%  High cholesterol = 43.8%  Alcohol = 43.3%  Unhealthy diet = 42.1%  Lack of exercise/unhealthy lifestyle = 29.0%  Obesity = 28.0%  Diabetes = 20.5%  Heart disease = 18.4%  Stress = 17.1%  Atrial fibrillation = 5.5%  Atherosclerosis = 2.6%  Others = 6.0% | 26.5% of respondents reported no knowledge of any warning signs or symptoms of stroke and the remaining 73.5% were able to list at least 1 sign or symptom correctly.  Perception of stroke warning symptoms among survey respondents (open-ended questions):  Paralysis/weakness = 31.0%  Dizziness/lack of balance = 30.7%  Headache = 30.4%  Altered consciousness = 20.9%  Sudden difficulty speaking = 12.1%  Blurred vision, loss of vision = 10.0%  Numbness, dead sensation = 7.7% |
| **Segura^(31)^ (2003) Spain** | Both | 59.6% of respondents were able to mention at least one stroke risk factor on open questioning.  Respondents’ knowledge of stroke risk factors on closed questioning:  Arterial hypertension = 92.0%  Alcohol = 90.3%  Smoking = 87.9%  Obesity = 87.4%  High cholesterol = 84.3%  Heart diseases = 83.5%  Lack of exercise = 76.9%  Emotions = 68.4% (false risk factor)  Advanced age = 68.3%  Diabetes = 59.0%  Gender = 27.7%  Arthritis = 24.0% (false risk factor)  Race = 6.7% | 32.6% of respondents were able to mention one or more signs of stroke on open questioning.  Respondents’ knowledge of stroke signs on closed questioning:  Paralysis/weakness = 88.4%  Sudden difficulty in speaking = 80.0%  Unconsciousness = 79.5%  Headache = 78.8%  Dizziness = 74.7%  Sudden loss of vision = 69.9%  Numbness = 68.6%  Lack of sensation = 67.9%  Sudden difficulty in swallowing = 47.2% |
| **Truelsen^(19)^ (2010) Denmark** | Closed | 72% of respondents included hypertension as one of the three most important risk factors for stroke. It is not clear what % of respondents could recognise at least one risk factor.  Risk factors prioritised by respondents from most prioritised to least prioritised (% not given):  Hypertension*  History of stroke  Hypercholesterolemia  Cardiovascular disease  Inheritance  Tobacco smoking*  Stress  Obesity  Inactivity  Alcohol  Diet  Diabetes*  *Classified as ‘major stroke risk factors’ in the study. | Participants were asked to identify the four most important symptoms of stroke from a list of symptoms. 10.2% of respondents identified four major stroke symptoms, 22.8% identified three symptoms, 40.3% identified two symptoms, 13.7% identified one symptom, and 2% identified no stroke symptoms. This meant that 98% of respondents were able to identify at least one symptom.  Order of identified stroke symptoms from most identified to least identified (% not given):  Aphasia*  Facial palsy*  Paresis*  Visual disturbances  Sensory disturbances*  Neck pain  Dizziness  Blindness  Chest pain  Neck stiffness  Abdominal pain  *Classified as ‘major stroke symptoms’ in the study. |
| **Vibo^(41)^ (2013) Estonia** | Closed | Respondents answered the following proposed stroke risk factors correctly, ranked in order of most correctly answered to least (exact % not given):  High blood pressure  Cigarette smoking  Insufficient physical activity  Excessive alcohol consumption  Low level of cholesterol in the blood (not stroke risk factor)  Cardiac arrhythmia  Diabetes  Drinking coffee (not stroke risk factor) = 45% answered incorrectly  No data given with regards to % of participants able to recognise at least one risk factor. | Respondents answered the following proposed stroke warning signs correctly, ranked in order of most correctly answered to least:  Speech disorder (dysphasia/aphasia) = 84%  Paralysis of a limb or side of the body = 83%  Paralysis of one side of the face, corner of the mouth drops down (no % given)  Sudden strong pain in the chest (not stroke symptom) = 29% answered incorrectly  Palpitation (not stroke symptom) = 29% answered incorrectly  Shortness of breath/dyspnoea (not stroke symptom) = 42% answered incorrectly  No data given with regards to % of participants able to recognise at least one warning sign. |
| **Vukovic^(33)^ (2009) Croatia** | Closed | Respondents’ knowledge of stroke risk factors:  Hypertension = 64.5%  Stress = 61.4%  Smoking = 58.9%  Elevated lipids = 53.1%  Obesity = 51.6%  Coagulation disorder = 46.9%  Alcoholism = 45.6%  Low-physical activity = 42.6%  Frequent headaches* = 39.6%  Elderly age = 38.9%  Cardiac diseases = 37.8%  Weather changes* = 34.5%  Drugs = 33.3%  Diabetes = 32.2%  Cervical pain* = 29.3%  Osteoporosis* = 6.3%  Bad digestion* = 5.0%  Gout* = 4.5%  Oral contraceptives* = 4.1%  Cholelithiasis* = 2.0%  *Not offered as a correct risk factor in the study.  No data given with regards to % of participants able to recognise at least one risk factor. | Respondents’ knowledge of stroke signs:  Speech disorder = 81.8%  Paraesthesia in half of body = 70.5%  Weakness of arm or leg = 54.9%  Unsteady gait = 54.7%  Malaise* = 52.6%  Monocular loss of vision = 43.5%  Persisting vertigo = 41.4%  Partial loss of vision* = 35.2%  Headache* = 25.9%  Cervical pain* = 17.5%  *Not offered as a correct warning sign in the study.  No data given with regards to % of participants able to recognise at least one warning sign. |
| **Vuletić^(34)^ (2006) Croatia** | Closed | 41.1% of subjects identified more than a half of correct stroke risk factors, 0.47% of subjects identified all and 0.47% identified none of them. This meant that 99.53% of subjects recognised at least one stroke risk factor.  Study subjects' knowledge of correct stroke risk factors:  Hypertension = 73.5%  Obesity = 70.0%  Hyperlipidaemia = 69.8%  Cigarette smoking = 67.0%  Physical inactivity = 66.0%  Stress = 65.0%  History of stroke or myocardial infarction = 50.9%  Cardiovascular disease = 50.0%  Heredity = 46.6%  Alcohol = 34.4%  Atherosclerosis = 26.4%  Older age = 21.6%  Diabetes mellitus = 18.8%  Male sex = 9.0% | 42% of subjects identified more than a half of correct warning signs of stroke, and 5% knew them all. 4.2% of subjects failed to recognise any of these, meaning 95.8% of subjects recognised at least one warning sign of stroke.  Study subjects’ knowledge of correct stroke warning signs:  Speech problems = 72.6%  One-sided weakness: 1 leg or 1 arm = 66.0%  Loss of balance or coordination = 65.0%  Tingling sensation - one-sided = 61.0%  Loss of consciousness = 47.6%  Dizziness (vertigo) = 34.9%  Impaired vision, diplopia, loss of vision in 1 eye = 33.0%  Sudden headache = 30.0% |

Please note that some studies made it clear where participants’ responses were correct, and others did not. The above table does not distinguish between correct and incorrect responses unless the authors of the original studies did.
