# Supplementary material for: Does socioeconomic position affect knowledge of the risk factors and warning signs of stroke in the WHO European region? A systematic literature review
Source: BMC Public Health. 2020 Sep 29;20:1473. doi: 10.1186/s12889-020-09580-x (PMC7526368; doi:10.1186/s12889-020-09580-x)
Supplement: Supplementary file 5 — Additional file 5. Details of results of individual studies by socioeconomic position. This file provides details of the breakdown of the results for individual studies by socioeconomic position. [file 12889_2020_9580_MOESM5_ESM.docx]

**Additional File 5: Details of results of individual studies by socioeconomic position**

| **First author (Date) Country** | **Risk factors** | | | | **Warning signs** | | | |
| --- | --- | --- | --- | --- | --- | --- | --- | --- |
|  | **No statistically significant differences by SEP?** | **Statistically better knowledge in higher SEP?** | **Statistically better knowledge in lower SEP?** | **Results** | **No statistically significant differences by SEP?** | **Statistically better knowledge in higher SEP?** | **Statistically better knowledge in lower SEP?** | **Results** |
| **Baldereschi^(16)^ (2015) Italy** |  | √ |  | Knowledge of >1 risk factor:  Elementary (1-8 years) OR = 1.00 (Ref)  High school (9-13 years) OR = 1.3 (1.0–1.8, p<0.05)  ≥ University (14+ years) OR = 1.6 (1.1–2.5, p<0.05) |  | √ |  | Knowledge of >1 warning sign:  Elementary (1-8 years) OR = 1.00 (Ref)  High school (9-13 years) OR = 1.7 (1.2–2.2, p<0.05)  ≥ University (14+ years) OR = 2.8 (1.9–4.3, p<0.05) |
| **Dominicis^(32)^ (2006) Italy** |  | √* |  | “Those with a lower level of education were less likely to know at least one correct risk factor”.  Statistical significance not reported. |  | √* |  | Individuals who had a good awareness of ischaemic stroke included those with the highest level of education.  Statistical significance not reported. |
| **Evci^(36)^ (2007) Turkey** |  | √ |  | Participants who gave ≥ 1 correct risk factor:  Education:  ≤ Primary school = 59.4%  ≥ Secondary school = 69.4% (p<0.05)  Perceived family income:  Low = 51.6%  High = 67.3% (p<0.001)  Occupation:  Non-worker = 62.1%  Currently employed = 69.4%.  ORs published but not clear if related to risk factors. |  | √ |  | Participants who gave ≥ 1 correct warning sign:  Education:  ≤ Primary school = 74.7%  ≥ Secondary school = 87.0%; OR = 1.35 (1.01–1.81, p<0.001)  Perceived family income:  Low = 71.4%  High = 82.5%; OR = 1.83 (1.33–2.50, p<0.001)  Occupation:  Non-worker = 78.1%  Currently employed = 87.1% (p<0.05). |
| **Hickey^(6)^ (2009) Republic of Ireland & Northern Ireland** | √ |  |  | Number of correct risk factors (RF):  ≤ Primary education: <2 RF = 37%; ≥2 RF = 63%;  ≥ Secondary level education: <2 RF = 36%; ≥2 RF = 64%.  “No significant associations found - on analysis of the association between demographic factors (which included education) and identification of correct stroke risk factors.” |  | √ |  | Number of correct warning signs (WS):  ≤ Primary education: <2 WS = 17%; ≥2 WS = 83%;  ≥ Second level education: <2 WS = 10%; ≥2 WS = 90%.  “Adjusted odds ratio analysis indicates that higher levels of knowledge of stroke warning signs were significantly associated with having second level education or greater (OR=1.9, p<0.001).” |
| **Lundelin^(29)^ (2012) Spain** | N/A | | | |  | √ |  | Prevalence of individuals with adequate stroke knowledge (defined as 4-6 symptoms correctly reported):  ≤ Primary school = 47.1% ± 1.0; OR = 1.00 (Ref)  High school = 69.7% ± 0.8; OR = 2.53 (2.11-3.04, p<0.05)  University = 77.6 ± 0.9; OR = 4.81 (3.80-6.09, p<0.05)  p for trend <0.001. |
| **Mata^(35)^ (2014) Austria, France, Germany, Italy, the Netherlands, Poland, Russia, Spain and UK** | N/A | | | |  | √* |  | Mean number of correct stroke symptoms recognised across the nine countries:  Low education = 3.4  Medium education = 4.0  High education = 4.4  Statistical significance not reported. |
| **Melnikov^(37)^ (2016) Israel** | N/A | | | |  | √ |  | “Spearman correlation analysis demonstrated that education (r = 0.194, p<0.001) and income (r = 0.183, p = 0.001), were associated with the number of stroke warning signs in Veteran Residents (VRs). Only education was significantly associated with the number of stroke warning signs in Immigrants From the Former Soviet Union (IFSUs) (r = 0.149, p = 0.043)”.  “An ordinal logistic regression analysis showed that in VRs, education was associated with the number of stroke warning signs: OR = 1.10 (1.01-1.19, p = 0.03). Among IFSUs, no significant associations were found between these variables.” |
| **Montaner^(7)^ (2001) Spain** | √  (for all except arrhythmia) | √  (for arrhythmia) |  | % correctly answering arrhythmia as a risk factor:  No school = 59%  Primary = 51%  High school = 44%  University = 56% (p<0.05)  For all other risk factors listed (hypertension, diabetes, smoking, alcohol, CHD) there was no statistical significance.  “When a multiple logistic regression analysis was used to identify factors independently associated with a ‘very good’ global stroke knowledge (knowing both signs and risk factors), university education remained significant: OR = 6.6 (3.0–14.7, p < 0.001).” |  | √ |  | % correctly answering dizziness as a warning sign:  No school = 48%  Primary = 61%  High school = 64%  University = 82% (p<0.05)  % correctly answering vision problem as a warning sign:  No school = 55%  Primary = 68%  High school = 78%  University = 88% (p<0.05)  For all other warning signs listed (aphasia, hemiparesis and headache) there was no statistical significance, but there was increasing knowledge as educational level increased.  “When a multiple logistic regression analysis was used to identify factors independently associated with a ‘very good’ global stroke knowledge (knowing both signs and risk factors), university education remained significant: OR = 6.6 (3.0–14.7, p < 0.001).” |
| **Moreira^(18)^ (2011) Portugal** |  |  | √* | “The less educated tended to recognise vascular risk factors more frequently.”  Statistical significance not reported. |  |  | √* | “The less educated tended to recognise warning signs more frequently.”  Statistical significance not reported. |
| **Müller‑**  **Nordhorn^(8)^ (2006) Germany** |  | √ |  | “In multivariable analyses, the maximum of naming four correct risk factors was significantly associated with a higher educational level.”  Correctly named risk factors OR (95% CI) and association with education (>10 years vs ≤10 years):  1 vs 0 = 1.06 (0.95-1.19)  2 vs 0 = 1.19 (1.08-1.29)^†^  3 vs 0 = 1.32 (1.22-1.43)^†^  4 vs 0 = 1.51 (1.37-1.67) ^†^.  ^†^significant results. | N/A | | | |
| **Neau^(38)^ (2009) France** | √ |  |  | “Level of education was not directly related to the knowledge of stroke risk factors.” | √ |  |  | “Level of education was not directly related to the knowledge of stroke warning signs.” |
| **Nedeltchev^(17)^ (2007) Switzerland** |  | √ |  | Good knowledge (defined as respondents who can name ≥5 risk factors and make ≤1 error):  Primary school = 4.9%  High school = 2.6%  University = 17.9% (p<0.001, difference between groups (Pearson χ^2^ test)).  Good knowledge of risk factors was related to university education (p<0.001). |  | √* |  | Good knowledge (defined as respondents who correctly recognise ≥3 symptoms and make ≤1 error):  Primary school = 58%  High school = 66%  University = 66%  Statistical significance not reported. |
| **Nordanstig^(39)^ (2014) Sweden** |  | √ |  | “Level of education showed association with knowledge of stroke risk factors in univariable analyses. These associations remained in multivariable analysis.”  Multivariate logistic regression analysis (knowledge of ≥3 risk factors):  Primary school OR = 1.00 (Ref)  Elementary school OR = 1.71 (1.12-2.61)  High school OR = 1.83 (1.33-2.52)  University OR = 2.80 (2.03-3.86) |  | √ |  | “High education was associated with knowledge of stroke symptoms.”  “Level of education showed association with knowledge of stroke symptoms in univariable analyses. These associations remained in multivariable analysis.”  Multivariate logistic regression analysis (knowledge of ≥3 stroke symptoms):  Primary school OR = 1.00 (Ref)  Elementary school OR = 2.14 (1.14-4.03)  High school OR = 1.37 (0.81-2.31)  University OR = 2.30 (1.38-3.80) |
| **Parahoo^(40)^ (2003) Northern Ireland** |  | √ |  | “Respondents with degree qualifications were aware of more risk factors (7.60, SD = 4.13) than those with no educational qualifications (5.28, SD = 5.87) (F = 10.02, d.f. = 3, p < 0.001).”  There is no mention of deprivation with regards to risk factor results. | √ |  |  | “Deprivation and educational qualifications had no statistical effect on the number of warning signs recognised.” |
| **Ramirez-Moreno^(30)^ (2015) Spain** |  | √ |  | Prevalence of respondents' knowledge of ≥1 vascular risk factor by demographic factor:  Educational level:  No study = 40.9%; OR Ref  Primary = 55.7%; OR = 1.8 (1.4-2.3, p<0.0001)  Secondary = 63.8%; OR = 2.5 (1.9-3.3, p<0.0001)  Tertiary = 72.5%; OR = 3.8 (2.9-5.0, p<0.0001).  Income €/year:  <10,000 = 51.4% OR Ref  10,001-20,000 = 64.4%; OR = 1.7 (1.4-2.2, p<0.0001)  20,001-30,000 = 69.5%; OR = 2.1 (1.6-2.9, p<0.0001)  30,001-40,000 = 69.8%; OR = 2.2 (1.4-3.5, p = 0.002)  >40,000 = 80.4%; OR = 3.9 (1.9-7.9, p<0.0001). |  | √ |  | Prevalence of respondents' knowledge of ≥1 warning symptom by demographic factor:  Educational level:  No study = 50.0%; OR Ref  Primary = 69.7%; OR = 2.3 (1.8-3.0, p<0.0001)  Secondary = 81.9%; OR = 4.6 (3.4-6.1, p<0.0001)  Tertiary = 86.4%; OR = 6.4 (4.7-8.7, p<0.0001).  Income €/year:  <10,000 = 63.2%; OR Ref  10,001-20,000 = 80.7%; OR = 2.4 (1.8-3.2, p<0.0001)  20,001-30,000 = 89.2%; OR = 4.8 (3.2-7.3, p<0.0001)  30,001-40,000 = 90.7%; OR = 5.7 (2.7-11.9, p<0.0001)  >40,000 = 92.2%; OR = 6.8 (2.4-19.2, p<0.0001). |
| **Segura^(31)^ (2003) Spain** |  | √ |  | “In the univariate comparison, home-based occupation (e.g. housewives, pensioners, unemployed and disabled people) (p<0.01) was associated with the inability to mention at least one stroke risk factor.” |  | √ |  | “Inability to mention at least one warning sign was associated with home-based occupation (e.g. housewives, pensioners, unemployed and disabled people) (p<0.01).” |
| **Truelsen^(19)^ (2010) Denmark** | √ |  |  | Educational level was not significantly associated with better knowledge of major stroke risk factors. | √ |  |  | Educational level was not significantly associated with better knowledge of major stroke symptoms. |
| **Vibo^(41)^ (2013) Estonia** |  | √ |  | Mean values of risk factor answers by educational level:  Basic/primary = 0.22  Secondary = 0.37  Vocational = 0.48  Higher = 0.44.  “Comparing the group with highest education to other educational levels, the results for the question on risk factors are significantly better than those of basic/primary education (all p<0.001).” |  | √ |  | Mean values of symptom answers by educational level:  Basic/primary = 0.08  Secondary = 0.39  Vocational = 0.44  Higher = 0.56.  “Comparing the group with highest education to other educational levels, the results for the question on symptoms are significantly better than those of basic/primary education (all p<0.001).” |
| **Vukovic^(33)^ (2009) Croatia** | √ |  |  | “Patients of lower education levels less frequently recognised physical inactivity as a risk factor for stroke”.  As there were 12 correct risk factor answers for respondents to choose from, this result is not substantial enough to say that people with lower SEPs have lower stroke risk factor knowledge, as there is no other mention of any differences in risk factor knowledge between different SEPs in this study. |  | √ |  | “Respondents with lowest education had the least knowledge regarding stroke signs (p<0.01).” |
| **Vuletić^(34)^ (2006) Croatia** |  | √ |  | “Statistically significant differences according to the level of education were found in the number of correct answers on stroke risk factors:  University level (p<0.001)  Two-year college (p<0.001)  Secondary school (p<0.001)  University degree vs secondary school (p = 0.009).” |  | √ |  | “Statistically significant differences according to the level of education were found in the number of correct answers on stroke warning signs:  University degree (p = 0.002)  Two-year college (p<0.001)  Secondary school (p = 0.005)  Primary school (no data).” |

√ = result of the individual study.

* = Authors of the individual study reported this result, albeit statistics were not reported. This may be because associations with SEP were not the primary purpose of their study. However, as they reported specific patterns in their results and have reported associations with SEP, the authors of this systematic review have acknowledged these.

N/A = not applicable as the study did not look at this aspect. Where cells are empty, this was not a result of the individual study.
